# Biomedical limits and emancipatory possibilities: a cultural analysis of traditional, complementary, and integrative medicine in Women's health

**DOI:** 10.3389/fgwh.2026.1757757

**Published:** 2026-04-08

**Authors:** Thabata Cristina Rosa Negrete, Antonio Rodrigues Ferreira Júnior

**Affiliations:** 1 School of Medical Sciences, Graduate Program in Public Health (Collective Health), State University of Campinas (Universidade Estadual de Campinas), Campinas, SP, Brazil; 2 Graduate Program in Public Health (Collective Health), Department of Nursing, State University of Ceará (Universidade Estadual do Ceará), Fortaleza, CE, Brazil

**Keywords:** complementary medicine, cultural studies, integrative medicine, public health, scoping review, traditional medicine, Women's health

## Abstract

Women's health care remains largely shaped by biomedical paradigms that prioritize reproductive functions and physiological outcomes, often sidelining subjective, social, and cultural dimensions. Traditional, Complementary, and Integrative Medicine (TCIM) is frequently presented as an alternative, yet its incorporation into women's health tends to follow biomedical logics of validation. The aim of this scoping review was to analyze how TCIM in women's health is configured in indexed scientific publications, combining Joanna Briggs Institute (JBI) methods with an interpretive lens from Cultural Studies and the Expanded Circuit of Culture. Because this approach analyzes scientific literature as a cultural artifact rather than aggregating clinical efficacy data, its application for establishing classical biomedical guidelines is limited; however, it provides a rigorous critical interpretation of the field. Searches were conducted in ten national and international databases with no language or year limits; of 2,474 records identified, 1,079 met the inclusion criteria. Data were mapped quantitatively and qualitatively, treating articles as cultural artifacts. Four discursive configurations emerged: “practice efficiency and efficacy” studies (45.6%; *n* = 492), focused on safety and physiological outcomes and often reducing complex systems to isolated techniques; descriptive and prevalence studies (41.5%; *n* = 448), which portrayed TCIM use mainly through sociodemographic profiles and consumer behavior; a category addressing institutionalization and methodological challenges (6.8%; *n* = 74), examining the structural and epistemological conditions for TCIM recognition within formal health systems; and a smaller group centered on emancipatory care and subjective experience (6.1%; *n* = 65), foregrounding autonomy, therapeutic bonding, and ancestral knowledges. Overall, the field is strongly dominated by biomedical translation, which sanitizes sensorial, spiritual, and relational dimensions and upholds epistemic hierarchies, yet counter-hegemonic narratives persist at the margins, indicating possibilities for epistemic pluralism and more emancipatory approaches to women's health.

**Systematic Review Registration:**

https://doi.org/10.17605/OSF.IO/7TZP2.

## 1 Introduction

The field of women's health operates as a territory of intense dispute, extending well beyond the boundaries of clinical practice. It is a space where cultural values, political interests, and epistemic hierarchies are constantly negotiated (1). Since the institutionalization of modern medicine, female bodies have been primarily addressed through reproductive functions—especially the uterus and breasts—while subjective, social, and affective dimensions are repeatedly rendered secondary or invisible (2, 3). Even with important advances driven by collective health movements and specific public policies, a biomedical, rationalist, and hospital-centered paradigm continues to dominate. Within this paradigm, natural life cycles such as menarche, menstruation, menopause and childbirth tend to be medicalized, and the complexity of health–disease processes is reduced to physiological metrics (4).

In this contested landscape, Traditional, Complementary, and Integrative Medicine (TCIM) occupies an ambivalent political and epistemic position. On the one hand, TCIM has been recognized by the World Health Organization (WHO) as a key resource for universal health coverage and patient-centered care (5). Policy documents underscore its strategic importance for planetary well-being and call for rigorous evidence produced through inclusive, multidisciplinary research capable of dialoguing with different rationalities of care systems rather than forcing them into reductionist models (6).

On the other hand, there remains a persistent gap between this political recognition and the way TCIM is actually incorporated into health systems, particularly in women's health. In many contexts, these practices are relegated to a secondary role, labelled as “alternative” or “complementary”. This semantic subordination is not trivial: it reflects and reinforces epistemic hierarchies that legitimize certain ways of knowing while marginalizing others, especially those that do not align with modern Eurocentric rationality (7). In this configuration, the marginalization of TCIM is not only a matter of access, but a form of epistemic violence that positions biomedicine as the sole arbiter of truth.

Complex therapeutic systems — such as Traditional Chinese Medicine, which operates through frameworks of Yin-Yang balance and Qi circulation developed over millennia; Ayurveda, a medical system originating in the Indian subcontinent over three thousand years ago that conceptualizes health through the equilibrium of vital energies (doshas); or Indigenous and Afro-diasporic healing practices grounded in specific territorial and spiritual cosmologies — are often reduced to isolated techniques or active compounds. This process of “translation” confers visibility and institutional acceptance, but at the cost of silencing the rationality, cultural context, and subjects that produce and sustain these knowledges.

From a Cultural Studies perspective, science must be scrutinized not as a neutral mirror of reality, but as a cultural practice that produces and circulates meanings. Scientific production functions as a cultural artifact, a dynamic arena where representations are constructed, contested, and stabilized (8, 9). Scientific articles do not simply report findings; they participate in the construction of “regimes of truth” that define which bodies are intelligible, which voices are authorized, and which experiences are deemed credible (10, 11). In this sense, any mapping of TCIM in women's health that limits itself to listing interventions and outcomes remains insufficient. It is necessary to move beyond a descriptive overview of “what works” to a critical interrogation of the cultural event of research itself: who is allowed to speak, how data are framed, and what remains unsaid in the process.

Despite the strength of hegemonic pressures, the field is not monolithic. “Insurgent knowledges” persist in the cracks of scientific production, particularly in studies that foreground autonomy, therapeutic bonding, and the re-appropriation of the body (12). These perspectives suggest that integrating TCIM into women's health goes far beyond incorporating new techniques. It involves rethinking the meanings of care, the conditions under which knowledge is produced, and the status assigned to different epistemologies. Integration, in this sense, is less about adding modalities to existing protocols and more about affirming epistemic pluralism that recognizes diverse ways of knowing as legitimate.

This study analyzes the contemporary cultural event of TCIM use in women's health as reflected in indexed scientific publications. Rather than focusing only on clinical effectiveness, it articulates the rigor of a scoping review with a cultural-analytical perspective grounded in the Expanded Circuit of Culture. This approach allows us to map the scientific production not only quantitatively, but also in terms of the meanings, silences, and “counter-productions” that shape the field. By listening to the margins of scientific discourse, the study seeks to illuminate both the limits imposed by biomedical rationality and the emancipatory possibilities of care that honors the complexity of women's health.

## 2 Materials and methods

### 2.1 Study design

This study was conceived and conducted as a Scoping Review, in accordance with JBI (Joanna Briggs Institute) guidance (14, 15). This approach was chosen because it is particularly suited to mapping the breadth and nature of evidence in emerging or heterogeneous fields, clarifying concepts, and identifying gaps in the literature. While traditional systematic reviews focus primarily on synthesizing evidence related to narrowly defined questions, the scoping review design allows for a broader and more flexible examination of the extent, characteristics, and distribution of research activities.

**Figure 1:**
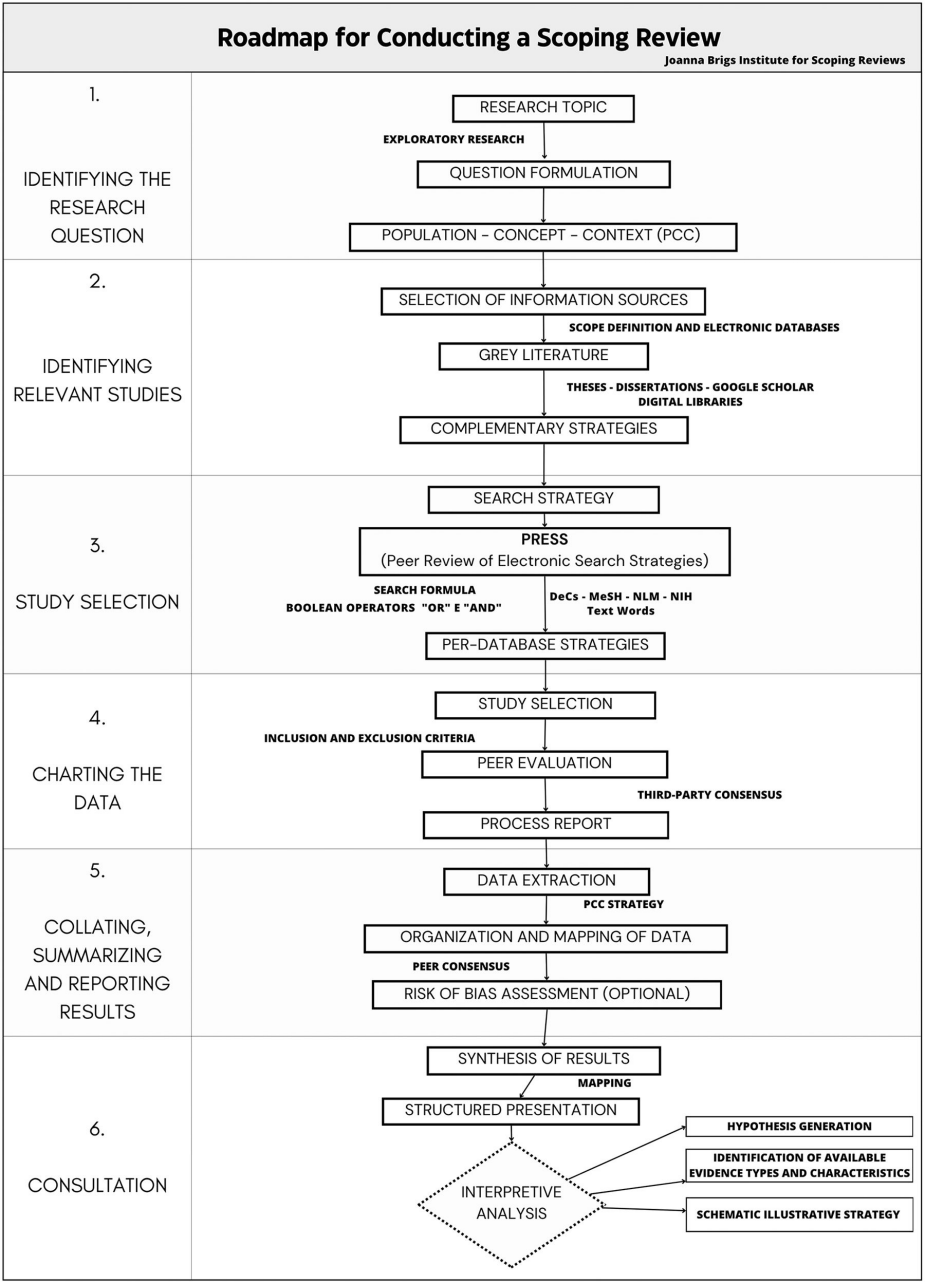
Roadmap developed by the author based on the joanna briggs institute (JBI) guidelines for conducting scoping reviews.

Moving beyond the mere aggregation of clinical outcomes, this review adopts an interpretive perspective grounded in Cultural Studies, understanding that research itself produces culture. Consequently, the JBI framework was utilized not just as a technical guide, but as a scaffold for critical analysis. The Expanded Circuit of Culture informed the entire process—from framing the research question to interrogating data and identifying silences (1). This deliberate departure from conventional scoping reviews means that, instead of categorizing studies by epidemiological design (e.g., randomized controlled trial, cohort, cross-sectional), we organized them by their discursive configurations—that is, how each article constructs and circulates meanings about women's health through TCIM practices. The methodological roadmap is presented in Figure 1.

To ensure transparency, reproducibility, and methodological rigor, the protocol was registered and made publicly available in the Open Science Framework prior to data extraction (DOI: 10.17605/OSF.IO/7TZP2).

### 2.2 Research question and eligibility criteria

The research question was formulated using the PCC mnemonic (Population, Concept, Context), aligning the scope of the review with the theoretical and political objectives of the study. The guiding question was: “What do scientific publications indexed in databases (Context) indicate about TCIM (Concept) in women's health (Population)?” (11).

Eligibility criteria were defined to include studies focusing on women's health without reproducing a strictly biomedical reduction of the female body. The population comprised women at any life stage (e.g., adolescence, pregnancy, menopause, aging) or in specific health conditions. Studies in which women's health appeared only as a subgroup within broader population analyses were excluded, to ensure that the corpus reflected investigations in which women's health experience was the primary analytical object rather than a secondary variable. Regarding the concept, TCIM was adopted in line with the World Health Organization's strategy (5), encompassing diverse practices such as acupuncture, herbal medicine, yoga, meditation, homeopathy, traditional midwifery, and other integrative therapies. The context included scientific publications (original research, reviews, and theoretical essays) indexed in academic databases. Grey literature, opinion pieces, case-reports, commentaries and conference abstracts were excluded, with the aim of focusing on validated scientific discourse. Studies addressing spirituality or religion as an isolated coping resource (e.g., Christian prayer as a strategy for coping with illness) without situating these practices within a TCIM framework were also excluded, as the object of this review is specifically the scientific production on TCIM.

### 2.3 Information sources and search strategy

To obtain a comprehensive overview of global scientific production on TCIM and women's health, the search was conducted in ten national and international electronic databases: PubMed, PubMed Central (PMC), Virtual Health Library (BVS/BIREME), Cumulative Index to Nursing and Allied Health Literature (CINAHL), Scopus, Web of Science, Embase, Cochrane Library, Psychological Abstracts (PsycINFO), and Scientific Electronic Library Online (SciELO—scielo.org). This selection was strategic to encompass not only biomedical literature, but also outputs from nursing, psychology, social sciences, and regional databases that are crucial for capturing research from the Global South (1).

The search strategy was constructed and peer reviewed in accordance with the Peer Review of Electronic Search Strategies (PRESS) guidelines (17), using a combination of controlled vocabulary (MeSH terms, DeCS descriptors) and free-text terms related to “Women’s Health” and “Complementary Therapies”. Boolean operators were adapted to the syntax of each database. No restrictions on language or publication year were applied, in order to maximize the retrieval of diverse epistemologies and avoid linguistic bias.

### 2.4 Data selection and processing

**Figure 2:**
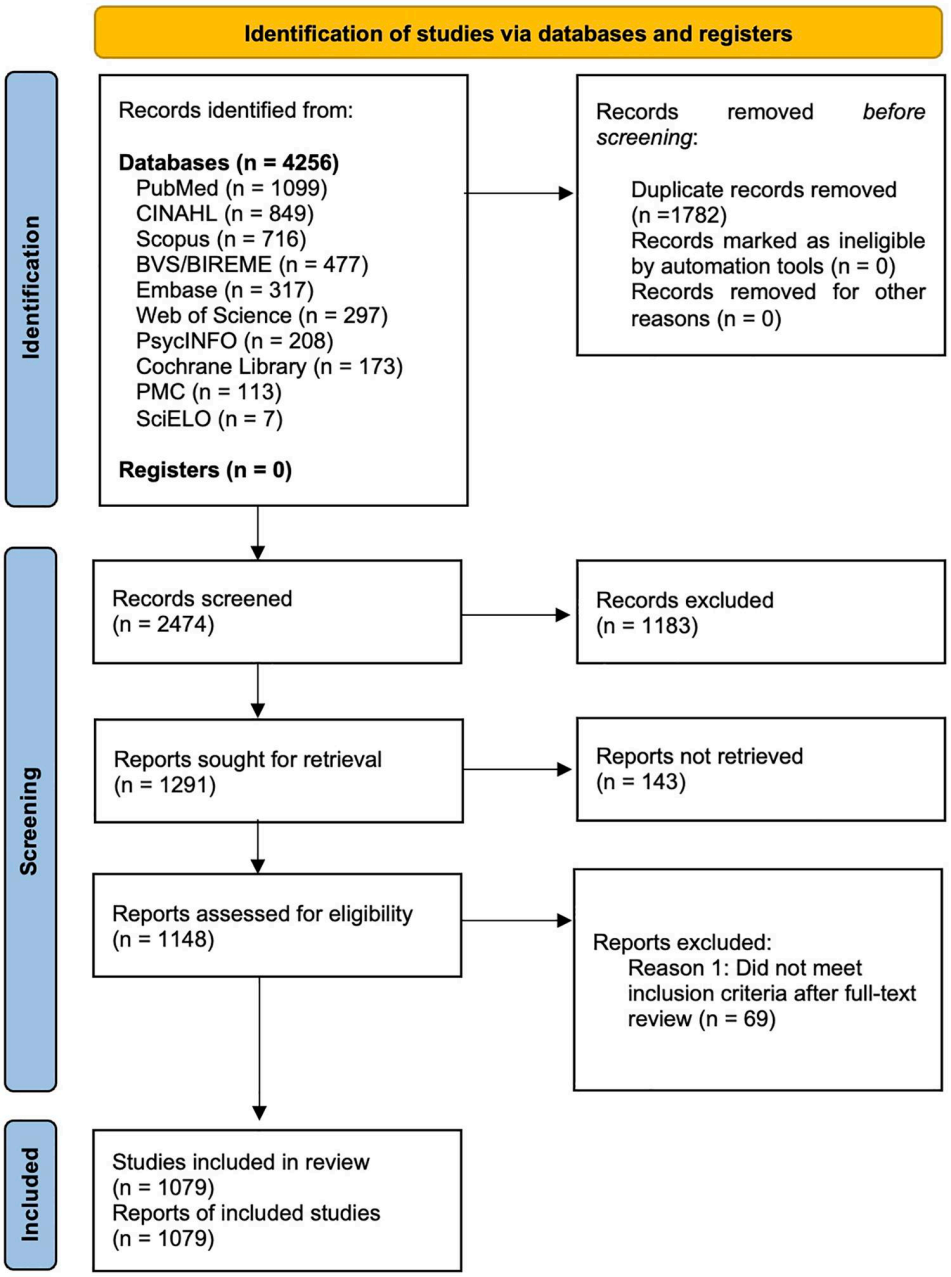
PRISMA 2020 flow diagram of the study selection process.

The selection process used Rayyan software to support blinded and independent screening by two reviewers. The steps following PRISMA 2020 and PRISMA-ScR guidelines (16) (Figure 2): identification of 2,474 unique records after removal of duplicates; screening of titles and abstracts against inclusion and exclusion criteria; and full-text assessment for eligibility. Discrepancies were resolved through consensus. Ultimately, 1,079 articles were included in the final analysis. Within the JBI framework for scoping reviews, the assessment of methodological quality is optional. Therefore, in accordance with the specific objective of our research, a formal risk of bias evaluation was not conducted.

### 2.5 Theoretical-analytical framework: the expanded circuit of culture

**Figure 3:**
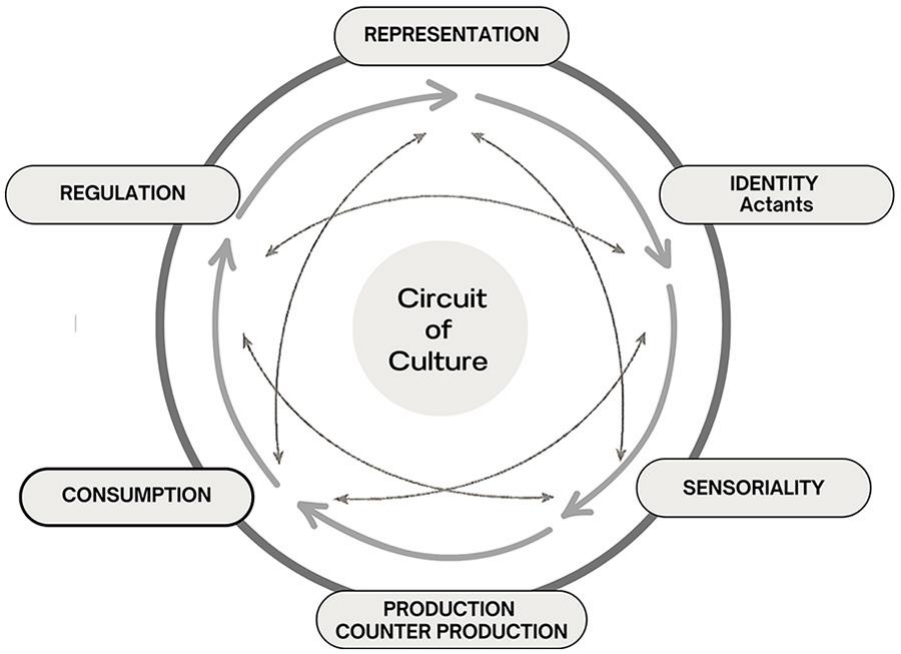
Original illustration of the expanded circuit of culture. Theoretical dimensions based on du Gay et al. (18) and Pereira (13), highlighting the dimensions of representation, identity (actants), production-counter production, consumption, regulation, and sensoriality.

Moving beyond descriptive mapping, this study adopted Cultural Studies as the overarching epistemological framework and the Expanded Circuit of Culture, proposed by Pereira (13), as the primary analytical tool. While the original model by du Gay et al. (18) was designed to analyze cultural artifacts such as consumer goods, Pereira's expanded version adapts and extends these insights to the health field, incorporating dimensions essential for examining complex care systems (Figure 3).

Data analysis treated scientific articles as cultural artifacts, not merely as repositories of information. The dimensions of Representation, Identity, Production, Counter-production, Consumption, Regulation, and Sensoriality were not applied as isolated categories, but as interconnected moments in the circulation of meanings. For example, the way TCIM is represented in a given article is inseparable from who is authorized to speak (Identity), how the study was designed and funded (Production), which practices are silenced or re-signified (Counter-production), and how scientific norms regulate what counts as valid evidence (Regulation). This relational reading enabled us to identify not only what is published, but also the power relations that sustain specific regimes of truth (1).

### 2.6 The researcher as an implicated interpreter

In keeping with the epistemological stance of Cultural Studies, the researcher is understood as an implicated subject, not an external, neutral observer (19). The author's trajectory in Collective Health and long-standing engagement with TCIM and feminist debates in health shaped both the formulation of the question and the interpretive choices. Rather than a bias to be eliminated, in line with interpretive traditions in Cultural Studies, the researcher's positionality was acknowledged as part of the analytical process and made explicit in order to enhance reflexivity and methodological transparency. Within this approach, the definition of inclusion criteria, the choice of databases, and the selection of descriptors were recognized as epistemic decisions rather than neutral procedural steps. Acknowledging that global indexing systems shape what is considered valid evidence, making these choices explicit is a requirement for transparency to avoid reproducing the silencing of non-hegemonic practices.

Thus, particular attention was paid to what remains unsaid in the corpus—the absences, omissions, and silences surrounding women's experiences and certain TCIM practices. The identification and interpretation of these silences were considered as central to the analysis as the extraction of explicit data.

## 3 Results

### 3.1 Overview of the corpus

The scoping review resulted in the inclusion of 1,079 articles out of 2,474 initially identified records. This corresponds to an inclusion rate of 43.6%, while 1,395 records were excluded for reasons such as: not addressing TCIM, not focusing on TCIM specifically (32.3%), or not focusing exclusively on women's health (7.2%), or addressing spirituality and religion outside the TCIM framework (2.9%). The high volume of exclusions highlights a persistent terminological ambiguity, particularly in the way “alternative” and “complementary” are used and how “complementary” is often conflated with non-TCIM biomedical adjuncts.

### 3.2 Selection and general characteristics of sources

Temporal analysis revealed a clear evolution in scientific production on TCIM in women's health. The earliest records were sparse, but there was substantial exponential growth in the last decade (2013–2023), which accounts for most of the corpus (*n* = 615 between 2003 and 2013, followed by concentrated output in subsequent years). This expansion coincides with global policy movements toward institutionalization, such as the WHO Traditional Medicine Strategy 2014–2023 (5), and national policies like Brazil's National Policy on Integrative and Complementary Practices (PNPIC) (20).

Geographically, publications were concentrated in high-income countries and emerging economies, with underrepresentation of regions where traditional practices are historically rooted but less indexed in mainstream databases. Thematically, the field appeared heavily skewed toward reproductive biology, with a dense cluster of studies on the pregnancy–childbirth–puerperium continuum and on menopausal transition. This distribution suggests that the scientific lens often replicates the biomedical fragmentation of the female body, privileging reproductive events while overlooking other dimensions of women's health. A synthesis of the four discursive configurations, including their prevalence and epistemological characteristics, is presented in Table 1.

**Table 1:** Configurations of scientific production on TCIM in Women’s health.

Discursive Configuration	Prevalence	Dominant Logic	Primary Focus & Outcomes	Representation of TCIM
Practice Efficiency and Efficacy	45.6% (*n* = 492)	Biomedical Validation	Physiological Outcomes	Isolated Technique
		Legitimacy is conditional upon translation into biomedical metrics of safety, efficacy, and pharmacological mechanisms.	Focus on symptom reduction (e.g., labor pain, nausea, hot flashes) measured by standardized scales and biochemical markers.	Complex systems (e.g., acupuncture, phytotherapy) are reduced to standardized protocols or active compounds, stripped of theoretical foundations.
Descriptive and Prevalence Studies	41.5% (*n* = 448)	Market & Consumerism	User Profile & Behavior	Lifestyle Commodity
		Health is framed as a commodity; usage is analyzed through behavioral and socioeconomic profiling (neoliberal logic).	Correlations between use and income/education; focus on “disclosure” to physicians and purchasing power rather than health meanings.	TCIM appears as an “alternative” product or adjunct service available in the private health market for those with access.
Institutionalization & Methodological Challenges	6.8% (*n* = 74)	Structural & Epistemological	Policies & Protocols	Peripheral Adjunct
		Conditions Focuses on structural barriers, demonstrating how systems regulate the recognition of legitimate practices and evidence.	Implementation of services, cost-effectiveness, and methodological debates on the tension between reductionist science and holistic systems.	TCIM is accommodated as peripheral “comfort measures” or optional services, so long as it does not disrupt established biomedical hierarchies.
Emancipatory Care and Subjective Experience	6.1% (*n* = 65)	Counter-Hegemonic	Autonomy & Agency	Ancestral Wisdom & Re-existence
		Epistemic pluralism that values subjective experience, relationality, and political dimensions of care.	Focus on reclaiming bodily control, therapeutic bonding, resistance to obstetric violence, and strengthening community ties.	TCIM is validated as a culturally situated rationality and a political resource for self-care and community resilience.

### 3.3 Practices, efficiency, and efficacy: the hegemony of biomedical validation

The largest category in this review, “Practice Efficiency and Efficacy”, encompassed 45.6% (*n* = 492) of the included studies. These articles investigated TCIM primarily through biomedical metrics of safety, efficacy, and efficiency. Within this category, the main subsets were Curative Practices (*n* = 277), Phytotherapy, and Mind–Body interventions. The central research question was predominantly framed around whether a specific intervention “works” according to allopathic standards, using randomized controlled trials (RCTs), quasi-experimental designs, and systematic reviews focused on physiological outcomes.

A closer examination of the Curative Practices subset (*n* = 277) showed that clinical targets were relatively homogeneous: reduction of labor pain, management of nausea and vomiting during pregnancy, relief of vasomotor symptoms during menopause, control of anxiety and depression, and management of chronic pain. Such outcomes were primarily measured using scales, biochemical markers, and standardized clinical endpoints.

In terms of interventions, acupuncture emerged as the most frequently studied practice, followed by herbal medicine, yoga, meditation, and homeopathic approaches. However, in many cases, acupuncture was reduced to a standardized protocol for symptom management rather than being approached as a holistic intervention rooted in traditional diagnoses. Similarly, phytotherapy was frequently split into isolated active compounds (e.g., soy isoflavones, phytoestrogens), which were tested as pharmacological equivalents of hormonal therapy. This process strips the herbal practice of its traditional, symbolic, and ritual dimensions, aligning it with the logics of drug development (21).

Mind–Body practices such as yoga, meditation, and relaxation techniques were generally investigated for their impact on stress reduction, sleep quality, or mood regulation. In these studies, openness to subjective experience was somewhat more visible, but still subordinated to standardized scales and psychometric instruments. Overall, the “Practice Efficiency and Efficacy” category reveals a field in which TCIM is largely evaluated according to biomedical criteria, while other dimensions of care are marginalized.

### 3.4 Descriptive and prevalence studies: the woman as consumer

Comprising 41.5% (*n* = 448) of the included studies, the second category grouped descriptive and prevalence analyses. In these articles, the primary objective was not to validate an intervention, but to characterize TCIM users based on sociodemographic variables such as age, income, education, and health insurance coverage, often with the intention of delineating a specific “user profile” (1).

Geographically, this category was disproportionately represented by studies from countries with consolidated private health sectors and strong consumer markets. In such contexts, TCIM is frequently portrayed as a commodity within a health marketplace. Women appear primarily as “consumers” making choices based on purchasing power, personal preferences, and perceived benefits. The notion of “access” is framed less as a right and more as a function of individual ability to pay or navigate health markets.

Several studies in this category also described the “communication” of TCIM use to biomedical providers, highlighting concerns about potential interactions between “alternative” consumption and conventional treatments. While these works contribute valuable epidemiological data, they rarely delve into the subjective motivations or cultural meanings attributed to TCIM practices. Instead, use patterns are interpreted largely through behavioral and consumerist lenses.

In this framing, the woman's agency is often reduced to the “freedom to choose” between therapeutic products on a shelf, detached from broader social, cultural, political, and commercial determinants of health. This reinforces an identity in which TCIM appears as an auxiliary commodity, rather than a plural therapeutic resource. Ultimately, the category mirrors neoliberal logics of health, in which care is commodified and legitimacy is tied to market demand (22).

### 3.5 Emancipatory care and subjective experience: counter-hegemonic narratives

In sharp contrast to the dominance of biomedical validation and consumerist approaches, a smaller corpus of studies (6.1%) was grouped in the category “Emancipatory Care and Subjective Experience”. These articles marked a clear epistemological shift, relocating the analytical focus from cure and consumption to lived experience, relationality, and the political dimensions of care.

Methodologically, this category was characterized by a predominance of qualitative designs, including ethnographies, phenomenological studies, in-depth interviews, and participatory approaches. These methods allowed women's voices, narratives, and embodied experiences to emerge as central data. TCIM practices were not evaluated solely as techniques for symptom relief, but as pathways for women to reclaim control over their bodies and life trajectories. This often occurred in contexts of resistance to obstetric violence, excessive medicalization, or neglect in conventional services.

Practices such as yoga, traditional midwifery, community-based integrative therapies, and Indigenous or Afro-diasporic healing systems were frequently analyzed as vehicles for autonomy and strengthening community ties. In these narratives, care was described as a collective process, intertwined with ancestral knowledge, spirituality, and territorial belonging. Women's subjective accounts and ancestral knowledges were treated as legitimate forms of evidence, directly challenging the epistemic hierarchies observed in the dominant categories (13).

### 3.6 Institutionalization and methodological challenges

A fourth configuration addressed the structural and epistemological conditions surrounding TCIM in women’s health. One subset, grouped under “Policies and Care Organization” (*n* = 44; 4.1%), examined the institutionalization of TCIM in formal health systems. These articles typically described the implementation of services in primary care, hospitals, and maternity units, as well as cost-effectiveness analyses for public health systems.

A critical reading of this subset revealed a recurring pattern: TCIM practices were frequently introduced as peripheral “comfort measures” or optional services, rather than as central components of therapeutic projects. Institutional arrangements tended to accommodate TCIM only insofar as it did not disrupt biomedical hierarchies or challenge existing power structures (1). This tension is evident, for instance, when institutionalized practices that meet biomedical standards (such as acupuncture) are readily offered in maternity wards, while ancestrally rooted practices intrinsic to a woman's cultural care itinerary (such as Afro-Brazilian leaf baths) remain invisible or prohibited.

A second subset, grouped as “Research and Protocols”, focused on methodological debates and protocol development. These studies underscored the challenges involved in applying reductionist scientific methods to complex, holistic systems—for example, designing double-blind RCTs for practices that rely on individualized diagnosis and therapeutic relationships. Authors discussed the tension between the need for methodological rigor and the risk of distorting TCIM to fit narrow evidence-based frameworks.

Taken together, these categories highlight that barriers to TCIM in women's health are not merely clinical or technical. They are structural, residing in how services are organized, how evidence is defined and produced, and how health systems regulate which practices are recognized as legitimate.

## 4 Discussion

This study set out not only to map scientific production on TCIM in women's health, but to interpret it as a cultural event situated within broader disputes over knowledge and power. By articulating the rigor of a scoping review with the Expanded Circuit of Culture, our analysis reveals a field marked by deep epistemic tension. The predominance of biomedical validation studies (45.6%) consolidates a “regime of truth” in which certain bodies and knowledges are considered legitimate while others remain peripheral or silenced (11). This asymmetry does not simply mirror the world; it reproduces and intensifies existing hierarchies by filtering traditional knowledges through a positivist sieve.

### 4.1 The limits of biomedical translation: regulation and representation

The heavy concentration of studies focused on efficacy, safety, and efficiency indicates that TCIM has largely been incorporated into the scientific circuit through translation into biomedical language. To be accepted, complex therapeutic systems rooted in diverse cosmologies and epistemologies are frequently reduced to isolated components or techniques and stripped of their theoretical foundations to fit the rigid metrics of randomized controlled trials (9).

From the standpoint of Representation, TCIM is often portrayed as a set of “adjunct treatments” or “alternative inputs” aimed at specific biological dysfunctions—such as using acupuncture solely to reduce nausea in pregnancy or supplementing soy isoflavones to manage menopausal symptoms—while the broader diagnostic and cosmological frameworks of Traditional Chinese Medicine or Ayurveda remain invisible (1). This operation is closely tied to Regulation: by requiring TCIM to “speak” the language of biomedicine, scientific norms reinforce the primacy of the physiological body over the lived body.

One of the most affected dimensions in this process is Sensoriality. In much of the literature, the patient’s feeling, therapeutic touch, atmosphere of rituals, and affective resonance are treated as “placebo effects” or methodological noise to be controlled. When these dimensions are systematically eliminated in the name of “objectivity”, what emerges is a sanitized version of TCIM that is acceptable to biomedical institutions, but distant from the practices' own rationalities and meaning-making processes. This sanitization helps maintain the biomedical monopoly over truth, integrating TCIM only as long as it behaves like a pharmaceutical intervention and, in so doing, perpetuates the coloniality of knowledge in women's health (8).

### 4.2 The woman as consumer vs. subject: identity and consumption

Applying the Identity and Consumption dimensions of the Expanded Circuit to descriptive and prevalence studies (41.5%) reveals a second important pattern. In this part of the corpus, women tend to be framed as consumers of TCIM, whose choices are explained mainly through sociodemographic variables and individual attitudes. The use of TCIM is frequently represented as a lifestyle decision accessible to those with higher income, education, and health insurance coverage.

In many of these studies, women's agency appears narrowed to a set of preferences within a health market offering products and services. The emphasis falls on the ability to choose between different therapies, often without attention to the structural conditions—such as racism, sexism, class inequalities, and territorial disparities—that shape these “choices”.

This framing is further reinforced by the way TCIM is portrayed as an adjunct commodity. The intervention is described as an optional complement to biomedical treatment, purchased according to purchasing power and cultural capital. The patient's subjectivity is reduced to adherence or non-adherence, satisfaction surveys, and willingness to pay. In this scenario, acts of seeking TCIM are interpreted as market behaviors rather than practices of care (22).

However, even within the corpus, these consumer framings are not uniform. A closer reading reveals representational displacements: the same act of seeking TCIM acquires different meanings depending on the social position of the women involved. In studies conducted with women in high-income, predominantly white contexts, TCIM use tends to be narrated as an empowered consumer choice — an assertion of autonomy within a marketplace of therapeutic options. In contrast, studies involving racialized or socioeconomically marginalized women — such as Mexican and Mexican-American communities — frame TCIM use as a response to the hostility or inaccessibility of mainstream biomedical services, where traditional systems are mobilized not as lifestyle preferences but as necessary resources for survival and cultural continuity. These displacements indicate that the identity constructed around the “TCIM user” in the scientific literature is not singular; it shifts according to the representational context in which it is produced, revealing how intersections of race, class, and territory shape the meanings attributed to the same practices.

Conversely, the minority of studies focused on “Emancipatory Care” (6.1%) offers another image. In these narratives, women who engage with TCIM are not simply consumers of services; they co-create care processes, negotiate meanings, and exercise agency in the face of institutional violence or neglect. Consumption itself is re-signified as a political act of self-care and “re-existence”, particularly in contexts of gendered and racialized oppression. The therapeutic bond moves away from a model of individualized service provision and approaches collective, relational, and territorial forms of care. The clash between these perspectives exposes that integrating TCIM into health systems is not merely a technical question, but a dispute over who the patient is: a market metric or a sovereign subject (13).

### 4.3 Counter-production and re-existence: the power of the margins

Perhaps the most significant finding of this review lies in the visibility of what appears at first sight to be marginal. The minority corpus that centers emancipatory care, ancestral knowledges, and community-based practices reveals the unintended effects and resistances generated within hegemonic systems. Despite intense pressure to medicalize TCIM and subsume it under the logics of evidence-based medicine, these studies document a vibrant counter-movement.

The persistence of articles addressing ancestral knowledge, spiritual practices, and collective healing spaces indicates that such knowledges continue to “re-exist” (12). They do not merely survive in parallel to biomedicine; they actively reconfigure meanings of health, illness, and care. In these narratives, the success of a TCIM intervention is not measured only by symptom suppression, but by the restoration of agency, validation of cultural identity, strengthening of community ties, and reconnection between body and territory.

In terms of the Expanded Circuit, these studies perform Counter-production: they subvert hegemonic representations and regulatory norms by insisting that women's experiences, affects, and sensoriality are not residual or anecdotal, but central to the production of health. The frequent sanitization of spirituality and affect through biomedical translation of these complex medical systems becomes meaningful in light of this minority corpus. Their existence demonstrates that it is possible to research TCIM without severing the connections between body, territory, and subjectivity (13).

### 4.4 Limitations and future directions

This study presents limitations inherent to its epistemological design that must be critically addressed. By explicitly adopting an interpretive approach grounded in Cultural Studies, this review departs from the positivist assumption of absolute neutrality often expected in classical scoping reviews. From the perspective of traditional clinical epidemiology, this framework might be perceived as a limitation in interpretability, as the findings are not intended to evaluate the universal physiological efficacy of TCIM practices or to generate standardized biomedical guidelines.

However, within this theoretical framework, acknowledging research as a situated practice does not imply subjective bias or ideological distortion. Rather, it refers to the systematic analysis of the macro-sociological power relations inherent in scientific production—specifically, the dynamics that dictate which epistemologies are legitimized as valid evidence and which are relegated to the margins. Acknowledging this positionality is a requirement for methodological transparency.

Furthermore, this macroscopic and cultural focus does not compromise methodological rigor or reproducibility. The interpretative path taken in this study was highly structured: the Joanna Briggs Institute (JBI) framework and PRISMA guidelines guaranteed the systematic tracking, selection, and blinding of the corpus, while the Expanded Circuit of Culture provided a standardized theoretical matrix for critical interpretation.

Therefore, while this theoretical lens restricts the study's utility for extracting purely biomedical efficacy metrics, it deliberately expands its critical capacity. Assuming these boundaries allows the research to move beyond basic data extraction to systematically expose epistemic hierarchies, generating valid and actionable reflections for institutional organization and cognitive justice in women's health.

Beyond these epistemological boundaries, this review acknowledges limitations that must be considered when interpreting the findings. First, the analysis is based on publications indexed in major databases, which inevitably privilege certain types of studies, languages, and countries. This may underrepresent local, community-based, and non-indexed productions, as well as grey literature and oral traditions where insurgent knowledges frequently circulate.

Furthermore, the databases themselves operate within a biomedical framework of classification and indexing, which tends to favor particular kinds of evidence and interventions. Consequently, the finding that only 6.1% of the studies focused on emancipatory care may not reflect an absence of these practices in reality, but rather the structural bias of global indexing systems and editorial criteria. Even with an inclusive search strategy and no language restrictions, there is a profound risk that more experimental, territorialized, or spiritual practices intrinsic to TCIM remain invisible to this mapping.

It is worth noting that certain practices—particularly acupuncture, phytotherapy, and specific mind-body techniques — appeared predominantly within the biomedical validation configuration, while traditional midwifery, community-based integrative therapies, and Indigenous and Afro-diasporic healing systems were almost exclusively represented within the emancipatory care configuration. However, we interpret this distribution not as an inherent property of the practices themselves, but as a consequence of their differential susceptibility to biomedical translation. Practices that can be isolated as discrete interventions or reduced to measurable active compounds are more readily co-opted by hegemonic scientific discourses, whereas practices embedded in relational, spiritual, and territorial logics resist such reduction and thus remain at the margins of the indexed literature.

Future research should engage with transdisciplinary methodologies that bridge clinical evidence and cultural experience, creating spaces where different epistemologies can dialogue on equal terms. This includes combining qualitative and quantitative approaches, co-producing research agendas with communities and practitioners, and valuing narrative, sensorial, and spiritual dimensions as legitimate sources of knowledge (7).

Ultimately, advancing the field requires moving beyond viewing TCIM as a set of “alternative” commodities. It calls for recognizing these practices as valid, culturally situated rationalities of care and as part of broader struggles for epistemic justice in women's health.

## 5 Conclusion

This study demonstrates that the integration of Traditional, Complementary, and Integrative Medicine into women's health research is far from a neutral process. It is a deeply political and epistemological dispute. Our analysis shows that, in much of the scientific literature, TCIM is incorporated through a logic of biomedical translation that grants legitimacy only when practices are stripped of their cultural, spiritual, and relational complexity. In this process, care systems are fragmented into isolated interventions and reduced to their effects on reproductive functions and physiological parameters, while subjective and sensorial dimensions—central to holistic care—are silenced (2, 13).

Yet, the persistence of “insurgent knowledges”, even in a marginalized corpus, points to another possible horizon. Studies that foreground ancestral knowledge, community-based practices, spiritualities, and emancipatory methodologies demonstrate that it is possible to research health without breaking the ties between body, territory, and subjectivity. They show women who use TCIM not only to alleviate symptoms, but to reconstruct meanings, resist institutional violence, and reclaim knowledge about their own cycles and lives. The existence of these narratives confirms that the margins of science are not empty; they are inhabited by practices of care that continue to thrive despite structural epistemicide (7, 12).

We argue that true integration requires more than adding acupuncture, yoga, or herbal medicine to existing biomedical protocols. It demands rethinking who produces evidence, which experiences are recognized as valid, and how different rationalities of care—biomedical, traditional, and popular—can coexist without being forced into a single hierarchy of value. For future research, the challenge is to adopt transdisciplinary methodologies that are sensitive to the sensoriality of care, attentive to affect, spirituality, and relationality, and capable of honoring the cultural contexts that make these practices meaningful to women (13).

To move beyond these limitations, this study proposes essential shifts for the future of TCIM in women's health. Journal editors and researchers must actively promote cognitive justice by valuing diverse methodological approaches that capture the subjective, relational, and political dimensions of healing. Furthermore, policymakers must ensure that the institutionalization of TCIM does not merely co-opt these practices into rigid biomedical and market-driven frameworks, but rather respects, protects, and integrates the plural epistemologies of users and traditional caregivers.

Listening to the margins of scientific production is not an exercise in nostalgia for “traditional” pasts, but an urgent ethical and political task. When TCIM practices are recognized not as exotic remnants or market products, but as culturally situated systems of knowledge, the possibility opens for a healthcare model that is more inclusive, equitable, and attuned to the complexity of women's lives. Because caring is not only about treating diseases; it is also an act of resistance.

## Data Availability

The original contributions presented in the study are included in the article/Supplementary Material, further inquiries can be directed to the corresponding author.
